# LncRNA SNHG15 acts as a ceRNA to regulate YAP1-Hippo signaling pathway by sponging miR-200a-3p in papillary thyroid carcinoma

**DOI:** 10.1038/s41419-018-0975-1

**Published:** 2018-09-20

**Authors:** Dong-Mei Wu, Shan Wang, Xin Wen, Xin-Rui Han, Yong-Jian Wang, Min Shen, Shao-Hua Fan, Zi-Feng Zhang, Qun Shan, Meng-Qiu Li, Bin Hu, Jun Lu, Gui-Quan Chen, Yuan-Lin Zheng

**Affiliations:** 1 Key Laboratory for Biotechnology on Medicinal Plants of Jiangsu Province, School of Life Science, Jiangsu Normal University, Xuzhou, 221116 PR China; 2 College of Health Sciences, Jiangsu Normal University, Xuzhou, 221116 PR China; 3 State Key Laboratory of Pharmaceutical Biotechnology, MOE Key Laboratory of Model Animal for Disease Study, Model Animal Research Center, Nanjing University, Nanjing, 210061 PR China

## Abstract

Over the past decade, lncRNAs have been widely reported in human malignant tumors, including papillary thyroid carcinoma. LncRNA SNHG15 has been validated to be a tumor facilitator in several types of malignancies. The present study focused on the biological role of SNHG15 in papillary thyroid carcinoma. Based on the result of qPCR analysis, we identified the strong expression of SNHG15 in human papillary thyroid carcinoma tissues and cell lines. Moreover, Kaplan–Meier method was utilized to analyze the internal relevance between SNHG15 expression and overall survival rate of patients with papillary thyroid carcinoma. Loss-of-function assays were designed and conducted to determine the inhibitory effects of silenced SNHG15 on the cell growth and migration in papillary thyroid carcinoma. The mechanical investigation indicated that SNHG15 upregulated YAP1 by sponging miR-200a-3p. Moreover, results of gain-of-function assays validated the anti-oncogenic function of miR-200a-3p in papillary thyroid carcinoma. Finally, results of rescue assays validated the function of SNHG15-miR-200a-3p-YAP1 axis in papillary thyroid carcinoma. YAP1 is known as an oncogene and a core factor of Hippo pathway. Here, we demonstrated that SNHG15 inactivated Hippo signaling pathway in papillary thyroid carcinoma. In summary, our findings demonstrated that SNHG15 serves as a competitively endogenous RNA (ceRNA) to regulate YAP1-Hippo signaling pathway by sponging miR-200a-3p in papillary thyroid carcinoma.

## Introduction

In recent years, the incidence of thyroid cancer is continuously increasing. Thyroid cancer has gradually become the commonest endocrine malignancy^1–3^. As the main subtype of thyroid cancer (approximately accounts for 80%), papillary thyroid cancer (PTC) mainly occurs in young women and children^4^. Although the prognosis of patients with early-stage PTC is favorable, the 5-year survival rate of patients with advanced PTC is only approximately 59%^5^. Therefore, it is urgent to find more effective therapeutic strategies.

With the development of human genome project, lncRNAs (longer than 200 nt) have attracted more and more attention of human beings. According to previous reports, we knew that lncRNAs could regulate progression of multiple cancers^6–11^. More and more lncRNAs have been studied in papillary thyroid carcinoma^12–16^. As a typical lncRNA, small nucleolar RNA host gene 15 (SNHG15) has been reported to be a tumor facilitator in colon cancer^17^, non-small cell lung cancer^18^, breast cancer^19^, pancreatic cancer^20^, and gastric cancer^21^. This study aims to investigate the specific function of SNHG15 in PTC progression. At first, SNHG15 was found to be upregulated in PTC tissues and cell lines. The prognostic value of SNHG15 for PTC patients was identified with Kaplan–Meier method analysis. Functional assays were designed and carried out in PTC cell lines to demonstrate the effects of SNHG15 on cell proliferation, cell apoptosis, cell migration, and epithelial–mesenchymal transition (EMT). Hereto, the oncogenic properties of SNHG15 were identified in PTC. Moreover, SNHG15 has been reported to act as a ceRNA in human cancers through modulating miRNA/mRNA axis^18,19^. Here, SNHG15 was considered as a potential ceRNA in PTC due to its cytoplasmic location. Next, miR-200a-3p was proved to be a target miRNA of SNHG15 in PTC cells through performing bioinformatics analysis, RIP assay, pull-down assay, and luciferase reporter assay. Furthermore, results of functional assays indicated that miR-200a-3p acted as a tumor suppressor in PTC. Similarly, YAP1 was verified to be a target of miR-200a-3p in PTC cells. SNHG15 could upregulate YAP1 through sponging miR-200a-3p. YAP1 is known as the downstream oncogene of Hippo pathway. Here, we further certified that SNHG15 promoted PTC progression through inactivating Hippo signaling pathway. Taken all together, SNHG15 acted as a ceRNA to modulate YAP1-Hippo pathway through binding with miR-200a-3p.

## Materials and methods

### Tissue samples

All tissue samples (PTC tissues, *n* = 92; corresponding normal tissues, *n* = 92) used in this study were obtained from Key Laboratory for Biotechnology on Medicinal Plants of Jiangsu Province, Jiangsu Normal University. The inclusion criteria of patients in this study were as follows: patients who were identified as PTC through pathological examination; patients who did not receive radiotherapy or chemotherapy before the surgery. The study acquired the approval of the Ethics Committee of Jiangsu Normal University. Written informed consent had been given by all participators. The samples were snap frozen in liquid nitrogen and maintained at −80 °C as soon as they were collected from patients.

### Cell culture

Four PTC cell lines (BHP5-16, BCPAP, K1, and BHP2-7), one normal thyroid epithelial cell line (Nthy-ori 3-1), and HEK-293T cell were purchased and obtained from American Type Culture Collection (Rockville, MD, USA). All cell lines used in this study were preserved in DMEM (GIBCO-BRL) medium (containing 10% FBS, 100 U/ml penicillin, and 100 mg/ml streptomycin) in a humidified atmosphere with 5% CO_2_ at 37 °C.

### Plasmid construction and transfection

To silence SHNG15, shRNA (Santa Cruz Biotechnology, USA) was constructed with sequence specifically targeted to SNHG15. miR-200a-3p mimics/inhibitor and negative controls were purchased from GenePharma (Shanghai, China). pcDNA3.1 (+) vector (GenePharma, Shanghai, China) was utilized to construct a pcDNA3.1-SNHG15/YAP1 vector. Transfections were finished by using Lipofectamine 2000 (Invitrogen, Carlsbad, CA, USA). The transfection efficiency was measured by applying qRT-PCR.

### qRT-PCR

Total RNA was extracted from PTC cell lines with Trizol solution (Invitrogen). PrimeScriptTM RTMaster Mix (TaKaRa, Dalian, China) was used for reverse transcription. qRT-PCR was carried out on a Light Cycler480 instrument (Roche, Basel, Switzerland) using SYBR Premix Ex Taq II (TaKaRa). The conditions of thermal cycling were illustrated as follows: 95 °C for 30 s followed by 40 cycles at 95 °C for 5 s and at 60 °C for 30 s. All primers were purchased from Invitrogen. GAPDH and U6 were taken as the internal control. The 2^−ΔΔCt^ method was utilized to calculate the relative expression levels.

### Cell proliferation assay

Two days after transfection, PTC cells were seeded on 96-well plates at a density of 5000 cells/well. The culture medium was regularly replaced. MTT dye (20 μl per well, Solarbio, Beijing, China) was added into each well at different time points (24, 48, 72, and 96 h) followed with incubation for 4 h at 37 °C. Next, the medium was removed, DMSO (150 μl per well; Sigma, USA) was added and mixed for 10 min. The absorbance (OD 570 nm) was observed and measured with Universal Microplate Spectrophotometer (Bio-Tek Instruments, Inc., Winooski, VT, USA).

### Colony formation assay

Three days after transfection, PTC cells were treated with trypsin (Solarbio, Beijing). Cells were placed in 6-well plates at a concentration of 500 cells/well and were cultured in RPMI 1640 (Invitrogen) containing 10% FBS (Gibco) under normal conditions for 14 days. To visualize and count the colonies, methanol and 0.5% crystal violet (Sigma) were separately used to fix and stain colonies.

### EdU assay

The proliferation ability of transfected cells was evaluated by applying EdU cell proliferation (Ribo, Guangzhou, China). Briefly, cells in the proliferating phase were treated with EdU for 2 h. After the cells were washed three times with 0.5 g/ml of PBS, DAPI (Invitrogen) nuclei counterstained cells for 10 min at room temperature in a dark room. Cells marked by DAPI were washed three times with PBS. At length, the number of marked cells were measured with the flow cytometer FACSCalibur DXP (BD Biosciences, Franklin Lakes, NJ, USA).

### Cell apoptosis assay

To analyze apoptosis condition of indicated cells, flow cytometry analysis was performed. According to the user guide, an Annexin V-fluorescein isothiocyanate (FITC)/propidium iodide (PI) kit (BD Biosciences, San Jose, CA, USA) was applied to stain cells. Apoptotic cells were analyzed by utilizing flow cytometer and CellQuest software version 0.9.3.1 (BD Biosciences).

### Cell migration assay

Transwell migration assay was conducted as previously described^22^. Briefly speaking, cells were placed in the upper chambers (Costar, Washington, DC, USA) which were filled with serum-free RPMI-1640 medium. On the other hand, the lower chambers were supplemented with complete medium. Twenty-four hours later, cells were well incubated, a cotton swab was used to remove cells stayed in the upper chamber. The cells that migrated into the membrane were fixed with 4% paraformaldehyde and stained with crystal violet.

### Immunofluorescence

Cells were cultured on glass slides and fixed with 4% formaldehyde for about 10 min. Next, 0.3% Triton X-100 was used for cell permeation. The slices were blocked by the goat serum for about 15 min at 37 °C. Subsequently, samples were incubated with anti-E-cadherin (1:80, Bioworld, MN, USA) and anti-N-cadherin (1:80, Bioworld, MN, USA) at 4 °C overnight and with goat TRITC-labeled secondary antibody (1:70, Bioworld, MN, USA) at 37 °C for 1 h. Meanwhile, DAPI (Genview Inc., Shanghai, China) was utilized for staining. At last, the fluorescence was visualized under a microscope (×400).

### Xenograft model

Briefly, the BCPAP cells stably transfected with sh-SHNG15 or sh-NC were injected into the 4-week-old female BALB/c nude mice at a density of 4 × 10^5^ cells. Afterwards, the nude mice were maintained in SPF condition. The tumor size and volume were checked and measured regularly. Thirty days after implantation, the mice were sacrificed. The tumors were resected for further analysis. The animal protocols complied with the rule of the ethics committee of Jiangsu Normal University.

### Subcellular fractionation assay

The cytoplasmic and nuclear extracts were extracted from PTC cells with NE-PER Nuclear and Cytoplasmic Extraction Reagents (Thermo Scientific, Waltham, MA, USA). RNAs isolated from the nucleus or cytoplasm were analyzed with RT-qPCR analysis to identify the levels of nuclear control (U6), cytoplasmic control (GAPDH), and lncRNA SNHG15.

### Luciferase activity assay

Based on the protocol of manufacturers, luciferase activity was analyzed with Dual-Luciferase Reporter Assay System (Promega). After required transfection, BCPAP and K1 cells were lysed in culture dishes which was added with lysis buffer. Varioskan Lux Detection System (Thermo Scientific) was utilized to determine relative luciferase activity which was normalized to Renilla.

### RIP

For RIP assay, PTC cell lines were co-transfected with pcDNA- MS2, pcDNA-SNHG15-MS2, and pBobi-MS2-GFP. Based on the user guide, RIP assay was conducted by utilizing anti-GFP antibody (Abcam, Cambridge, UK) as well as a MagnaRIP RNA-Binding Protein Immunoprecipitation Kit (Millipore, Bedford, MA, USA).

For anti-AGO2-RIP assay, BCPAP and K1 cell lines were transfected with the pMIR vector expressing a negative control or miR-200a-3p, miR-18b-5p, miR-141-3p (Vigenebio Company, Shandong, China). Two days later, RIP assay was carried out by using anti-AGO2 antibody (Millipore).

### Western blot analysis

Western blot analysis was performed as previously reported^23^. In brief, cell lysates were prepared with RIPA buffer (Beyotime Biotechnology, Shanghai, China). Next, BCA Kit (Solarbio, Beijing) was used to quantify the protein concentrations. Samples were segregated by 10% SDS-polyacrylamide gel (Solarbio, Beijing) and transferred onto a PVDF membrane (Millipore). Subsequently, the immunoblot was incubated with anti-E-cadherin (1:1000, ab76055), anti-β-catenin (1:1000, ab32572), anti-N-cadherin (1:1000, ab76057), anti-Vimentin (1:1000, ab8978), anti-GAPDH (1:1000, ab8245) at 4 °C overnight and with Goat Anti-Mouse IgG H&L (HRP, 1:2000, ab6789) at 37 °C for 1 h. The primary and secondary antibodies were purchased from Abcam (Cambridge, UK). Next, ECL (Millipore) was applied for chemiluminescence detection. The immunoblot signal was quantified with Image J software.

### Statistical analysis

SPSS 17.0 software (SPSS, Chicago, IL, USA) was utilized for statistical analyses in this study. Differences between two groups were evaluated with Student’s *t*-test (two-tailed). Survival curve was generated with Kaplan–Meier method. Correlations among SNHG15, miR-200a-3p, and YAP1 were analyzed with Spearman rank correlation. Each experiment was conducted more than twice. Experimental results are presented as mean ± SD. Data was considered as statistically significant when *P* value was less than 0.05.

## Results

### Upregulation of SNHG15 predicted unfavorable prognosis of PTC patients

**Fig. 1:**
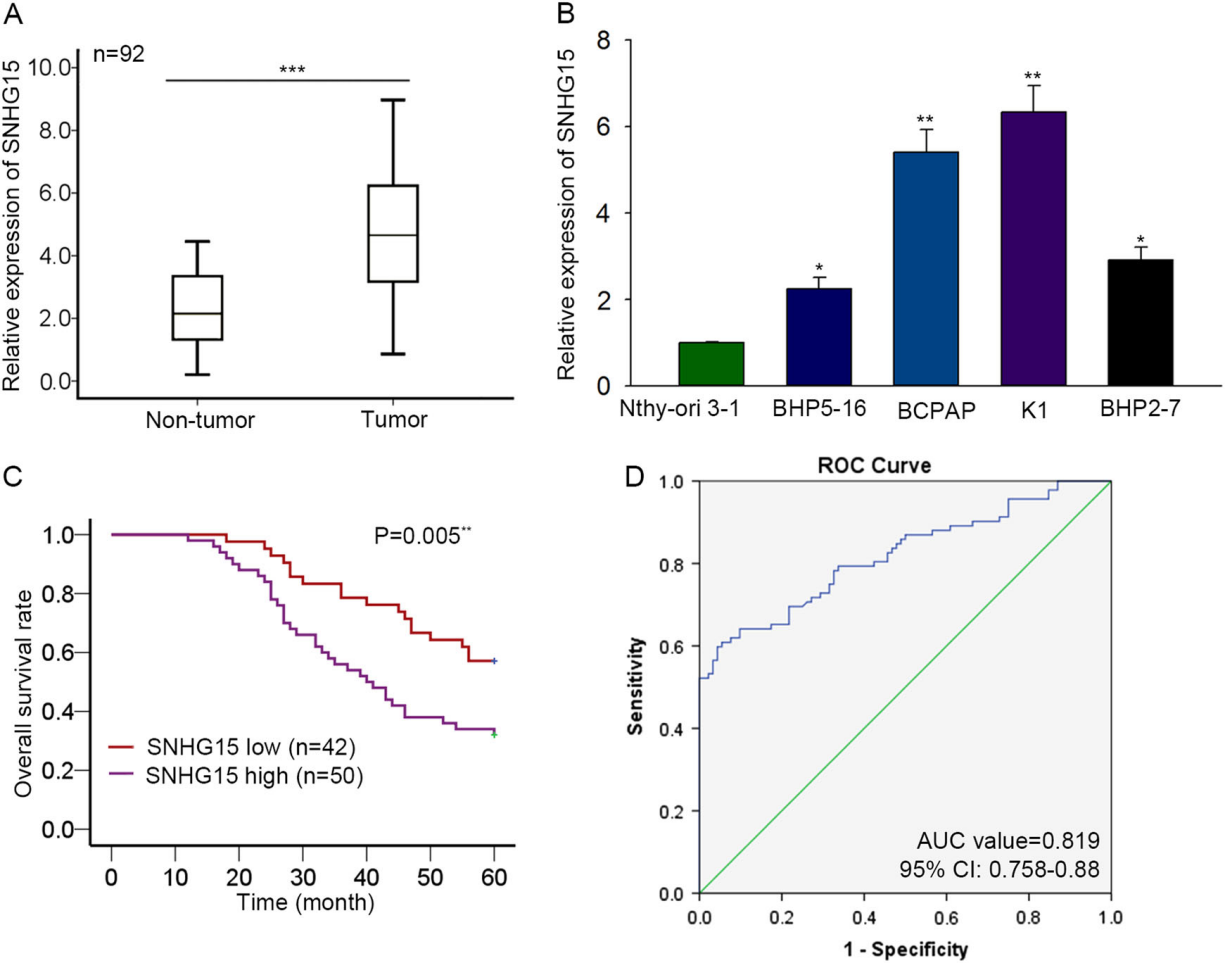
Upregulation of SNHG15 predicted unfavorable prognosis of PTC patients. **a**, **b** The expression level of SNHG15 was separately examined in tissues (PC tissues and adjacent normal tissues) and cell lines (one normal cell line and four PC cell lines). Results were obtained by using qRT-PCR analysis. **c** Survival curve was generated and analyzed with Kaplan–Meier method. **d** The receiver-operating characteristic curve for lncRNA SNHG15 in the prognosis of papillary thyroid cancer. *P < 0.05, **P < 0.01, ***P < 0.001

Here, we applied a qRT-PCR analysis to investigate the expression pattern of SNHG15 in PTC tissues and cell lines. The adjacent non-tumor tissues and normal cell line were used as the control group. As expected, SNHG15 was upregulated in PTC tissues (Fig. 1a, *P* < 0.001, *t* = −8.760). Consistent with this, the level of SNHG15 was higher in PTC cell lines (Fig. 1b). The level of SNHG15 in the normal cell line Nthy-ori 3-1 was taken as the control, the level of SNHG15 was significantly higher in BHP5-16 (*P* = 0.015, *t* = −8.103), BCPAP (*P* = 0.005, *t* = −14.176), K1 (*P* = 0.004, *t* = −15.239), and BHP2-7 (*P* = 0.013, *t* = −8.777). Next, the median value of SNHG15 expression was used as the cutoff value, all PTC samples were divided into two groups (SNHG15 high and SNHG15 low). The correlation between SNHG15 expression and the clinical features of PTC patients was analyzed. It was uncovered that higher expression of SNHG15 was closely related with gender (*P* = 0.024), larger tumor size (*P* = 0.030), advanced TNM stage (0.002), and positive lymph node metastasis (*P* < 0.001) (Table 1). To verify the prognostic value of SNHG15 for PTC patients, Kaplan–Meier method was carried out. High expression of SNHG15 was negatively correlated with the overall survival rate of PTC patients (Fig. 1c, *P* = 0.005). Furthermore, the ROC curve was generated for further analysis. The AUC value was 0.819 (95% CI = 0.758–0.88) (Fig. 1d), indicating the prognostic value of SNHG15 for PTC patients.

**Table 1:** Correlation between SNHG15 expression and clinical features of PTC patients (*n* = 92)

Variable	SNHG15 expression		*P*-value
	Low	High	
**Age**			
<45	30	36	0.952
≥45	12	14	
**Gender**			
Male	25	18	0.024*
Female	17	32	
**Extra thyroidal extension**			
Negative	18	13	0.088
Positive	24	37	
**Tumor size**			
≤1	28	22	0.030*
>1	14	28	
**TNM stage**			
I/II	24	13	0.002**
III/IV	18	37	
**Lymph node metastasis**			
Negative	23	9	<0.001***
Positive	19	41	
**Nodular Goiter**			
Negative	33	30	0.056
Positive	9	20	

Low/high by the sample median. Pearson *χ*^2^ test

*P < 0.05, **P<0.01 or ***P<0.001 was considered to be statistically significant

### Downregulation of SNHG15 inhibited cell growth and promotes cell apoptosis

**Fig. 2:**
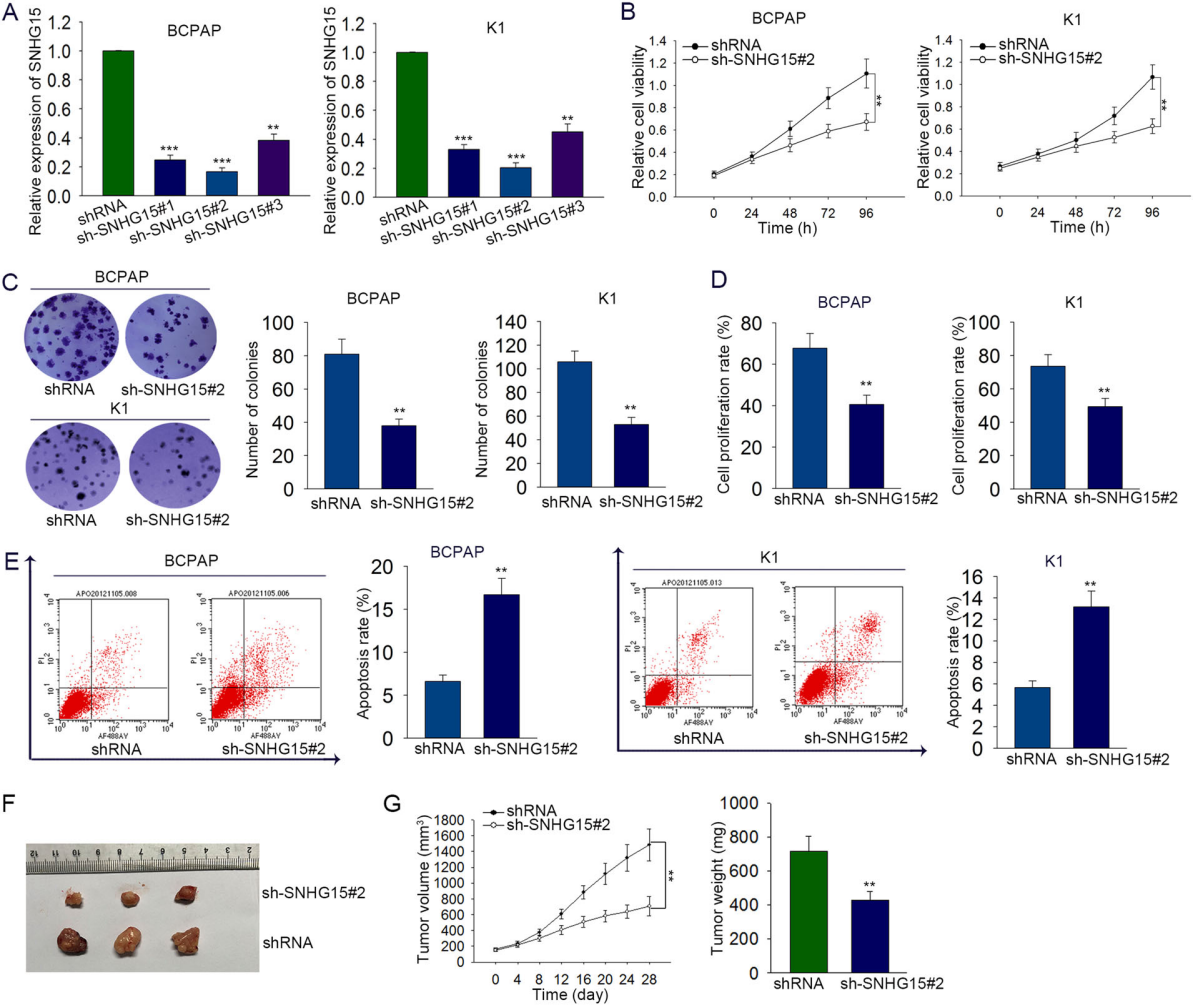
Downregulation of SNHG15 inhibited cell proliferation and promotes cell apoptosis. **a** SNHG15 was silenced in BCPAP and K1 cells by transfecting with specific sh-SNHG15. shRNA was used as a control. **b**, **c** MTT and colony formation assays were conducted to determine the influence of SNHG15 on cell proliferation. **d** EdU assay was further performed to validate the effects of silenced SNHG15 on cell proliferation. Scale bar = 200 μm. **e** Apoptosis of SNHG15-downregulated PTC cells was examined with flow cytometry analysis. **f**, **g** Xenograft model was constructed to demonstrate the effect of sh-SNHG15 on the tumor growth. **P < 0.01, ***P < 0.001

To evaluate the underlying function and mechanism of SNHG15 in the pathological process of PTC, functional assays were designed and carried out in PTC cell lines. According to the data of Fig. 1b, SNHG15 was expressed strongest in BCPAP and K1 cells. Therefore, we designed loss-of-function assays in BCPAP and K1 cells. Before the functional assays, SNHG15 was silenced in BCPAP and K1 cells by transfecting with SNHG15-specific shRNAs (sh-SNHG15#1, sh-SNHG15#2, sh-SNHG15#3) (Fig. 2a). shRNA was used as the negative control. Forty-eight hours later, qRT-PCR analysis was used to detect the transfection efficiency. The optimal efficiency was observed in sh-SNHG15#2 group (*P* < 0.001, *t* = 55.654). Therefore, it was chosen for subsequent experiments. Subsequently, MTT and colony formation assays were conducted to determine the influence of SNHG15 on cell proliferation. As illustrated in Fig. 2b, c, cell proliferation was markedly suppressed by silenced SNHG15. The result of MTT assay reflected that the proliferation was markedly suppressed in BCPAP (*P* = 0.001, *t* = 4.067) and K1 (*P* = 0.008, *t* = 3.079) cells transfected with sh-SNHG15. Moreover, colony formation of BCPAP (*P* = 0.007, *t* = 7.513) and K1 (*P* = 0.02, *t* = 8.476) cells was inhibited by sh-SNHG15. EdU assay was further performed to validate the inhibitory effects of silenced SNHG15 on cell proliferation. As a result, the proliferation of BCPAP (*P* = 0.008, *t* = 5.690) and K1 (*P* = 0.010, *t* = 5.004) cells was obviously inhibited (Fig. 2d). Additionally, cell apoptosis was examined with flow cytometry analysis. The enhanced apoptosis rate was shown in BCPAP (*P* = 0.006, *t* = −8.606) and K1 (*P* = 0.006, *t* = −8.60565) cells transfected with sh-SNHG15 (Fig. 2e). To monitor the effect of silenced SNHG15 on the tumor growth in vivo, SNHG15-silenced BCPAP cells were injected into the nude mice. The results suggested that sh-SNHG15 efficiently suppressed tumor growth (Fig. 2f), leading to smaller tumor size and volume (Fig. 2g).

### Knockdown of SNHG15 inhibited cell migration and EMT progress in PTC

**Fig. 3:**
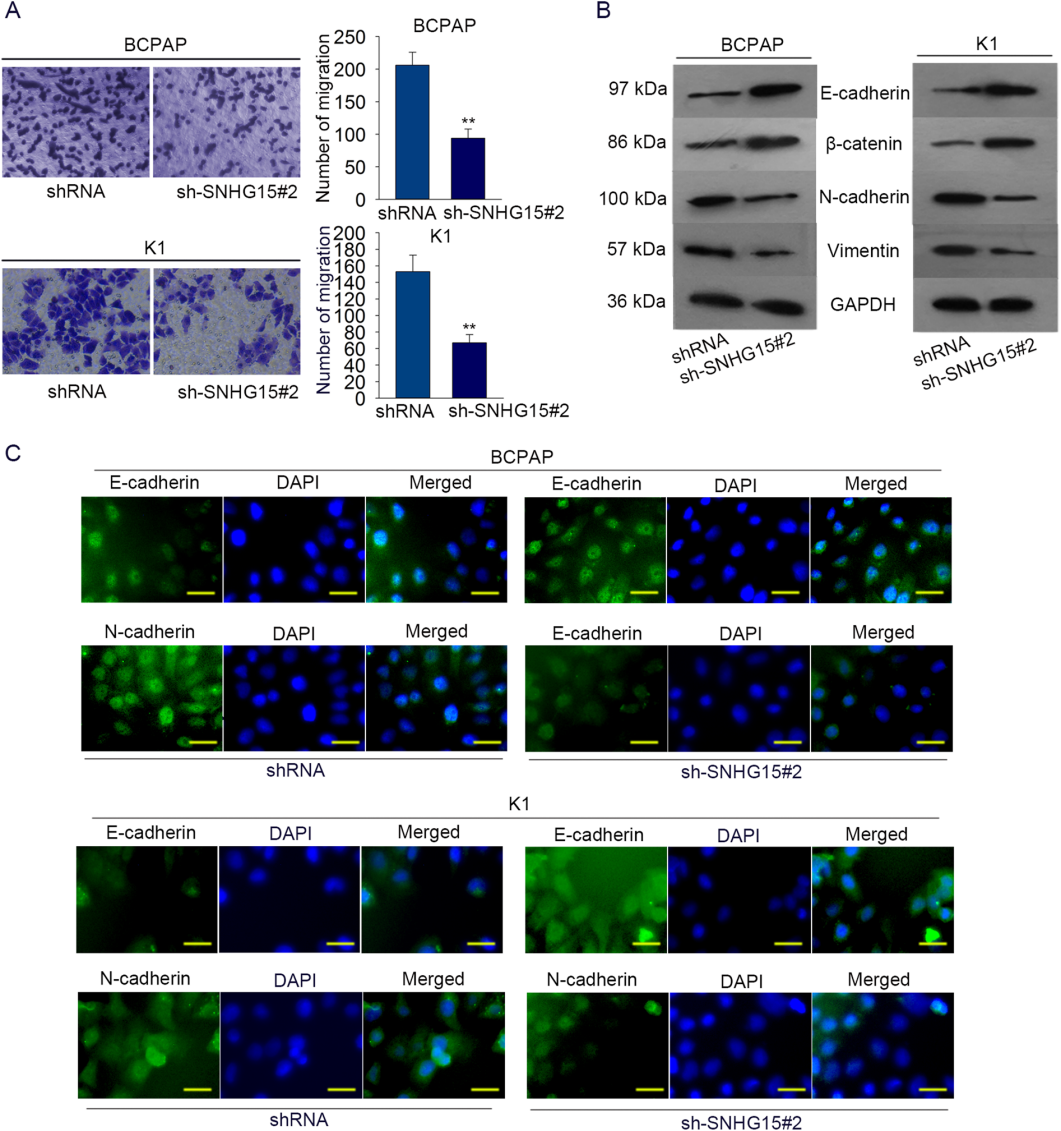
Knockdown of SNHG15 inhibited cell migration and EMT progress in PTC. **a** The influence of silenced SNHG15 on cell migration was detected by applying transwell assay. **b**, **c** Western blot analysis and immunofluorescence were separately utilized to analyze the impacts of SNHG15 knockdown on EMT progress in PTC cells. Scale bar = 200 μm. **P < 0.01

According to the above findings, SNHG15 could promote cell proliferation and inhibit cell apoptosis. Here, we further detect the influence of silenced SNHG15 on cell migration and EMT progress. Based on the result of transwell assay, we found that the migratory ability of BCPAP (*P* = 0.002, *t* = 7.899) and K1 (*P* = 0.007, *t* = 6.731) cells was inhibited by sh-SNHG15 (Fig. 3a). In addition, western blot analysis and immunofluorescence were utilized to analyze the impacts of SNHG15 knockdown on EMT progress. As shown in Fig. 3b, the levels of epithelial markers (E-cadherin, β-catenin) were enhanced, while the levels of mesenchymal markers (N-cadherin, Vimentin) were obviously reduced. The results of immunofluorescence were consistent with that of western blot analysis (Fig. 3c). Therefore, we confirmed that SNHG15 could improve cell migration and EMT progress.

### miR-200a-3p is a target of SNHG15

**Fig. 4:**
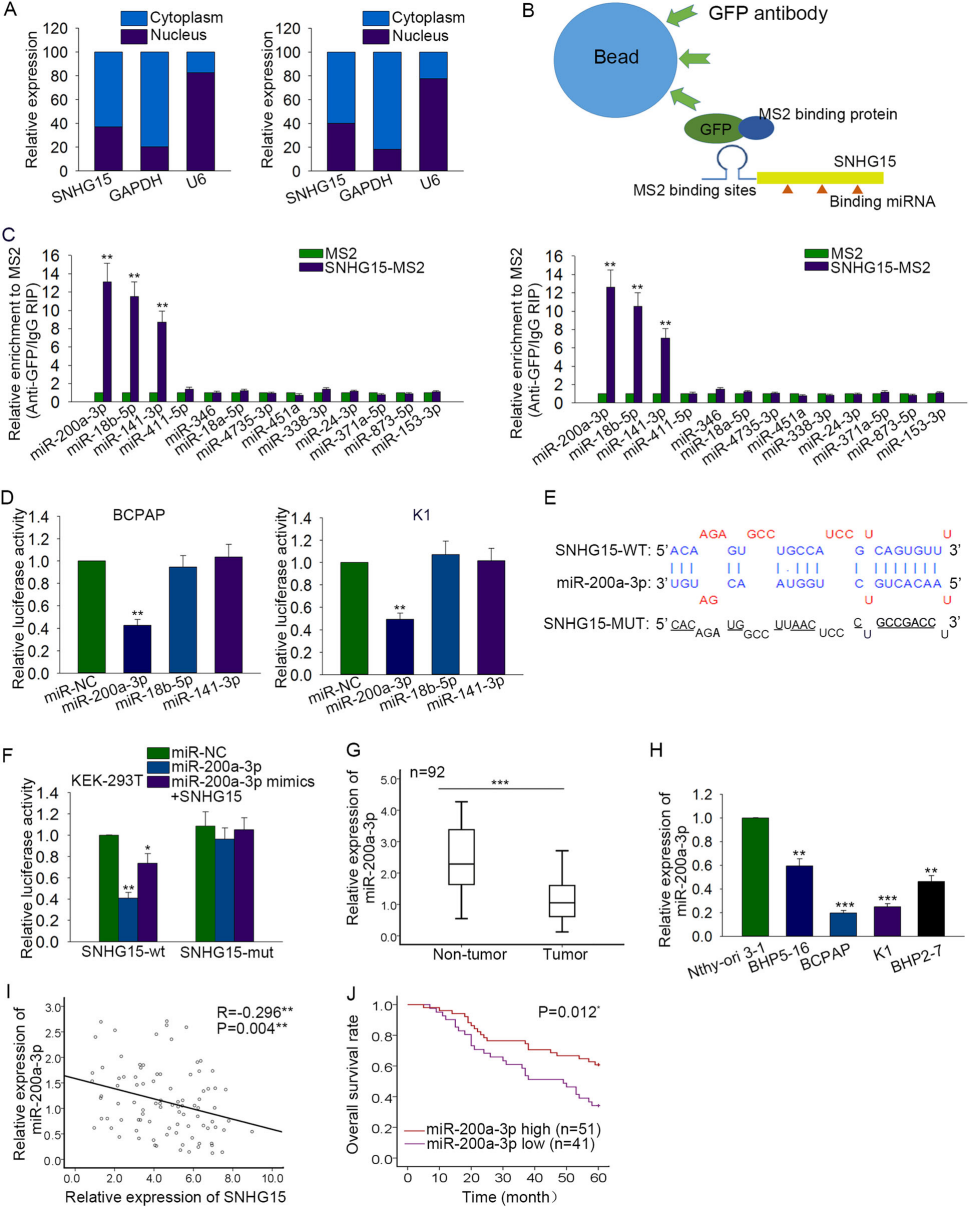
miR-200a-3p is a target of SNHG15. **a** The specific location of SNHG15 was identified in PTC cells through performing subcellular fractionation assay. **b** MS2-RIP assay was designed and conducted. **c** The interaction between 13 candidate miRNAs and SNHG15 was determined with RIP assays. **d** A luciferase reporter containing SNHG15 was constructed to prove the combination between SNHG15 and three miRNAs. **e** The binding sites between wild type SNHG15 or mutated SNHFG15 and miR-200a-3p were predicted by using bioinformatics analysis. **f** Dual luciferase reporter assay was carried out in HEK-293T cell to confirm the combination between SNHG15 and miR-200a-3p mimics. **g**, **h** qRT-PCR analysis was applied to determine the expression pattern of miR-200a-3p in PTC tissues and cell lines. **i** The correlation between SNHG15 and miR-200a-3p was obtained and analyzed through using Spearman’s correlation analysis. **j** The correlation between miR-200a-3p expression and the overall survival of PTC patients was analyzed by Kaplan–Meier analysis. *P < 0.05, **P < 0.01, ***P < 0.001

SNHG15 has been certified to act as a ceRNA in human cancers. Here, we hypothesized that SNHG15 exert oncogenic function through acting as a ceRNA in PTC cells. Firstly, the localization of SNHG15 was identified by performing subcellular fractionation assay. Obviously, SNHG15 was mainly located in the cytoplasm of PTC cells (Fig. 4a). Next, 13 candidate miRNAs were uncovered from starbase (http://starbase.sysu.edu.cn/). The interaction between miRNAs and SNHG15 was determined with RIP assays (Fig. 4b). Compared with SNHG15 immunoprecipitates obtained from cells treated with empty vector (MS2) or control IgG, those obtained from BCPAP and K1 cell lines were obviously enriched with three miRNAs, namely, miR-200a-3p (*P* = 0.009, *t* = −10.2134), miR-18b-5p (*P* = 0.008, *t* = −11.438), and miR-141-3p (*P* = 0.008, *t* = −10.924) (Fig. 4c). Furthermore, a luciferase reporter vector containing SNHG15 was constructed to prove the combination between SNHG15 and three miRNAs above. We found that only the ectopic expression of miR-200a-3p could decrease the luciferase activity of SNHG15 reporter vector in BCPAP (*P* = 0.003, *t* = 19.558) and K1 (*P* = 0.004, *t* = 15.874) cells (Fig. 4d). Subsequently, the binding sites between wild type SNHG15 or mutated SNHFG15 and miR-200a-3p were predicted by using bioinformatics analysis (Fig. 4e). As we all know, HEK-293T cell is easier to transfect. The experiment conducted in HEK-293T cell could help to reduce the derivation of experimental results. It was uncovered that the decreased luciferase activity of wild type SNHG15 (SNHG15-WT) caused by miR-200a-3p mimics (*P* = 0.003, *t* = 19.654) was recovered by SNHG15 overexpression (*P* = 0.036, *t* = 5.150) (Fig. 4f). However, the luciferase activity of mutated SNHG15 (SNHG15-MUT) was almost not changed. Subsequently, miR-200a-3p was uncovered to be downregulated in PTC tissues and cell lines (Fig. 4g, h). Accordingly, the expression correlation between SNHG15 and miR-200a-3p was proved to be negative (Fig. 4i, *P* = 0.004). Kaplan–Meier method was then used to analyze the correlation between the expression of miR-200a-3p and the overall survival of PTC patients. The result manifested that patients with lower level of miR-200a-3p had poorer overall survival than patients with higher level of miR-200a-3p (Fig. 4j, *P* = 0.012).

### miR-200a-3p inhibited PTC progression

**Fig. 5:**
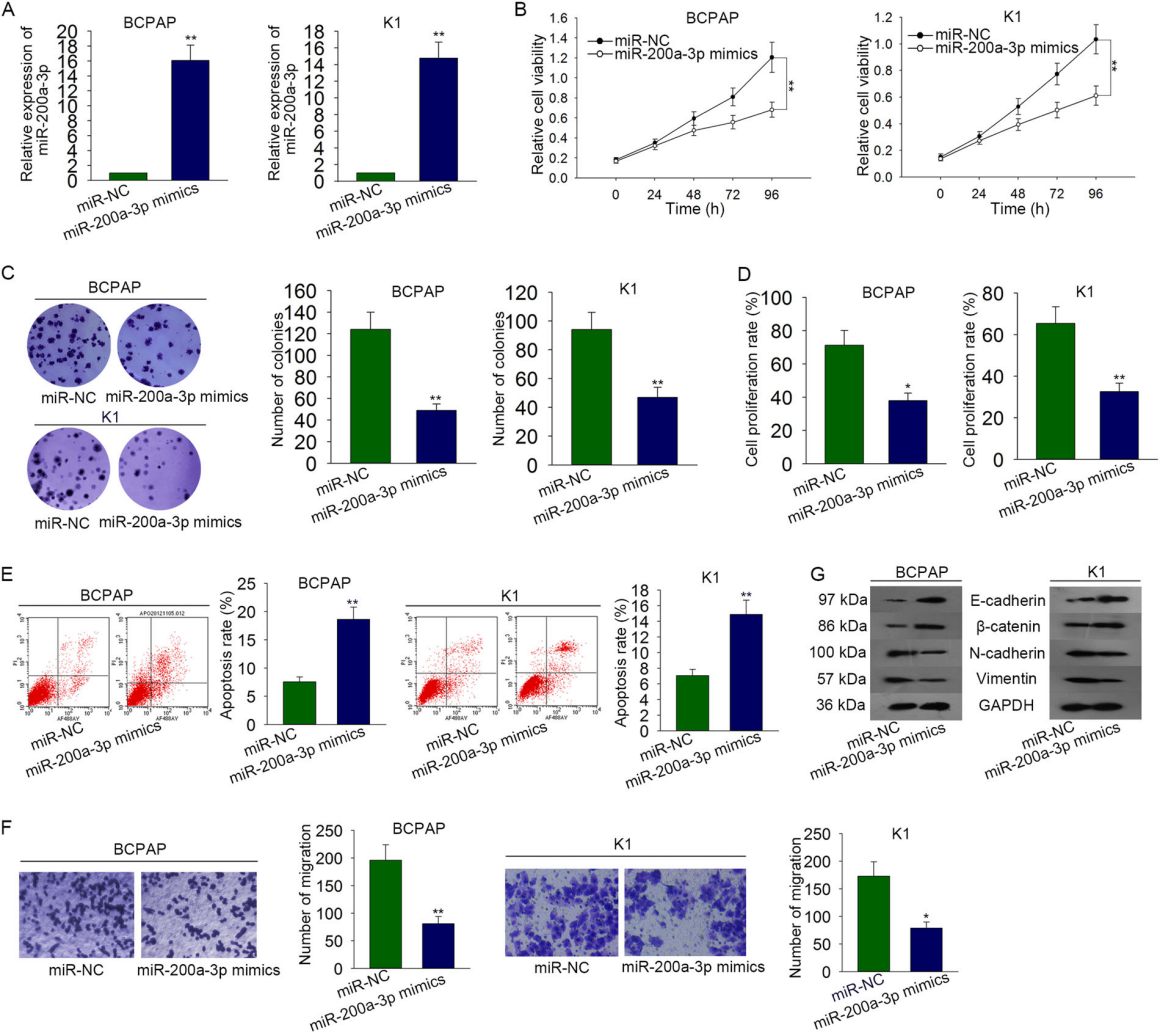
miR-200a-3p inhibited PTC progression. **a** miR-200a-3p was overexpressed with miR-200a-3p mimics in BCPAP and K1 cells. miR-NC was taken as the control group. **b**–**d** MTT assay, colony formation assay, and EdU assay were separately carried out to identify the influence of miR-200a-3p mimics on PTC cell proliferation. EdU: Scale bar = 200 μm. **e** Cell apoptosis rate was assessed by performing flow cytometry analysis. **f** Cell migration ability was evaluated with transwell assay. **g** The effect of miR-200a-3p mimics on EMT progress of PTC cells was analyzed through detecting the EMT-related proteins with western blot analysis. *P < 0.05, **P < 0.01

According to the previous study, we knew that miR-200a-3p is a tumor suppressor in some types of malignancies. Nevertheless, the specific function of miR-200a-3p in PTC progression is still marked. miR-200a-3p was overexpressed with miR-200a-3p mimics in BCPAP (*P* = 0.006, *t* = −12.525) and K1 (*P* = 0.007, *t* = −12.082) cells (Fig. 5a). Forty-eight hours later, the transfection efficiency was measured by qRT-PCR analysis. miR-NC was taken as the control group. Likewise, MTT assay, colony formation assay, and EdU assay were carried out to identify the influence of miR-200a-3p mimics on PTC cell proliferation. Cell proliferation was observably inhibited by miR-200a-3p mimics (Fig. 5b–d). However, the apoptosis of BCPAP (*P* = 0.006, *t* = −8.412) and K1 (*P* = 0.009, *t* = −6.804) cells was promoted by miR-200a-3p mimics (Fig. 5e). According to the result of transwell assay, the migratory capability of BCPAP (*P* = 0.008, *t* = 6.574) and K1 (*P* = 0.014, *t* = 5.755) cells was obviously suppressed by miR-200a-3p overexpression (Fig. 5f). Finally, the result of western blot analysis revealed that EMT progress was inhibited in miR-200a-3p-overexpressed PTC cells (Fig. 5g).

### SNHG15 upregulated YAP1 through targeting miR-200a-3p

**Fig. 6:**
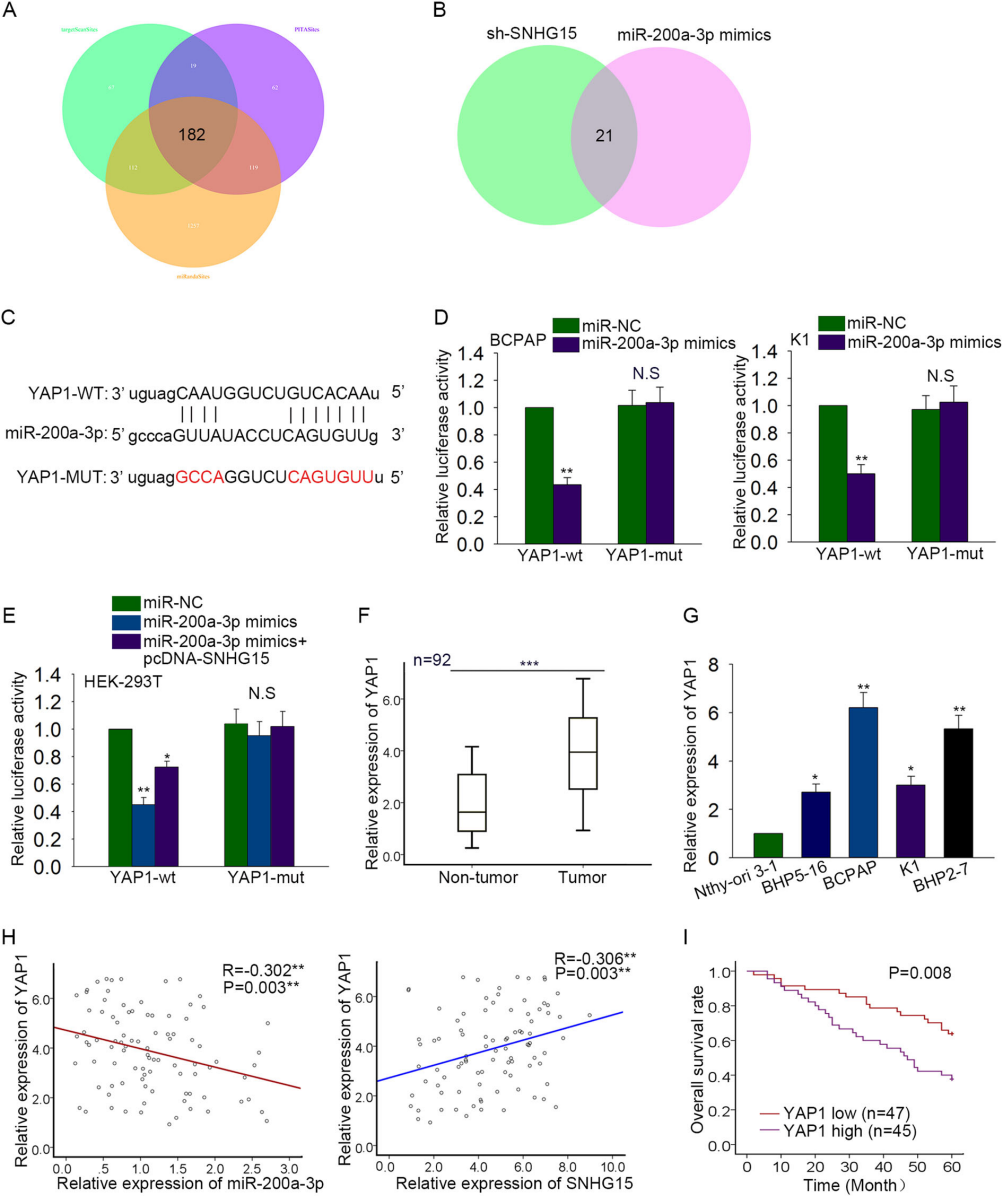
SNHG15 upregulated YAP1 through targeting miR-200a-3p. **a** The potential target mRNAs of miR-200a-3p were predicted by three bioinformatics tools (targetScan, picTar, and miRanda). **b** The levels of 182 candidate mRNAs were tested in response to sh-SNHG15 and miR-200a-3p mimics. **c** The binding sites between 3′UTR of YAP1 and miR-200a-3p were predicted by using bioinformatics analysis. **d** Luciferase reporter vectors were constructed to demonstrate the combination between miR-200a-3p and YAP1. **e** Dual luciferase reporter assay was conducted in HEK-293T cell which was co-transfected with miR-200a-3p mimics and pcDNA-SNHG15. **f**, **g** The expression levels of YAP1 were tested in both PTC tissues and cell lines through using qRT-PCR. **h** Spearman’s correlation method was utilized to analyze the relevance between miR-200a-3p and YAP1 as well as between SNHG15 and YAP1. **i** The prognostic value of the miR-200a-3p expression for PTC patients was identified with Kaplan–Meier method. *P < 0.05, **P < 0.01, ***P < 0.001; N.S. no significance

To support the ceRNA hypothesis, the target mRNA of miR-200a-3p must be found. 182 mRNAs were found to be the potential targets of miR-200a-3p in accordance with the prediction of three bioinformatics tools (targetScan, picTar, and miRanda) (Fig. 6a). Next, the levels of all these 182 mRNAs were examined in response to SNHG15 knockdown or miR-200a-3p overexpression. As a result, there are only 21 mRNAs that could be downregulated by both sh-SNHG15 and miR-200a-3p mimics (Fig. 6b). Among these 21 mRNAs, YAP1 is a famous oncogene, which is the downstream gene of Hippo signaling pathway. Therefore, YAP1 was chosen to do the next study. The binding sites between 3′UTR of YAP1 and miR-200a-3p were predicted (Fig. 6c). Similarly, luciferase reporter vectors were constructed to demonstrate the combination between miR-200a-3p and YAP1. The luciferase activity of wild type YAP1 (YAP1-WT) was markedly reduced in BCPAP (*P* = 0.003, *t* = 19.040) and K1 (*P* = 0.006, *t* = 12.868) cells transfected with miR-200a-3p mimics, while that of mutated YAP1 (YAP1-MUT) was not changed (Fig. 6d). Next, HEK-293T cell was co-transfected with miR-200a-3p mimics and pcDNA-SNHG15. The result of luciferase reporter assay suggested that the decreased luciferase activity of YAP1-WT caused by miR-200a-3p mimics (*P* = 0.003, *t* = 18.027) was increased again by pcDNA-SNHG15 (*P* = 0.008, *t* = 11.262) (Fig. 6e). Similarly, the luciferase activity of mutated YAP1 was not changed. The high expression levels of YAP1 were detected in both PTC tissues and cell lines (Fig. 6f, g). At last, the negative relevance between miR-200a-3p and YAP1 (*P* = 0.003) as well as the positive relevance between SNHG15 and YAP1 (*P* = 0.003) were analyzed (Fig. 6h). Similarly, the prognostic value of YAP1 for PTC patients was identified with Kaplan–Meier method. Consistent with SNHG15, higher level of YAP1 predicted poorer prognosis for PTC patients (Fig. 6i, *P* = 0.008).

### Function of SNHG15-miR-200a-3p-YAP1 axis in PTC progression

**Fig. 7:**
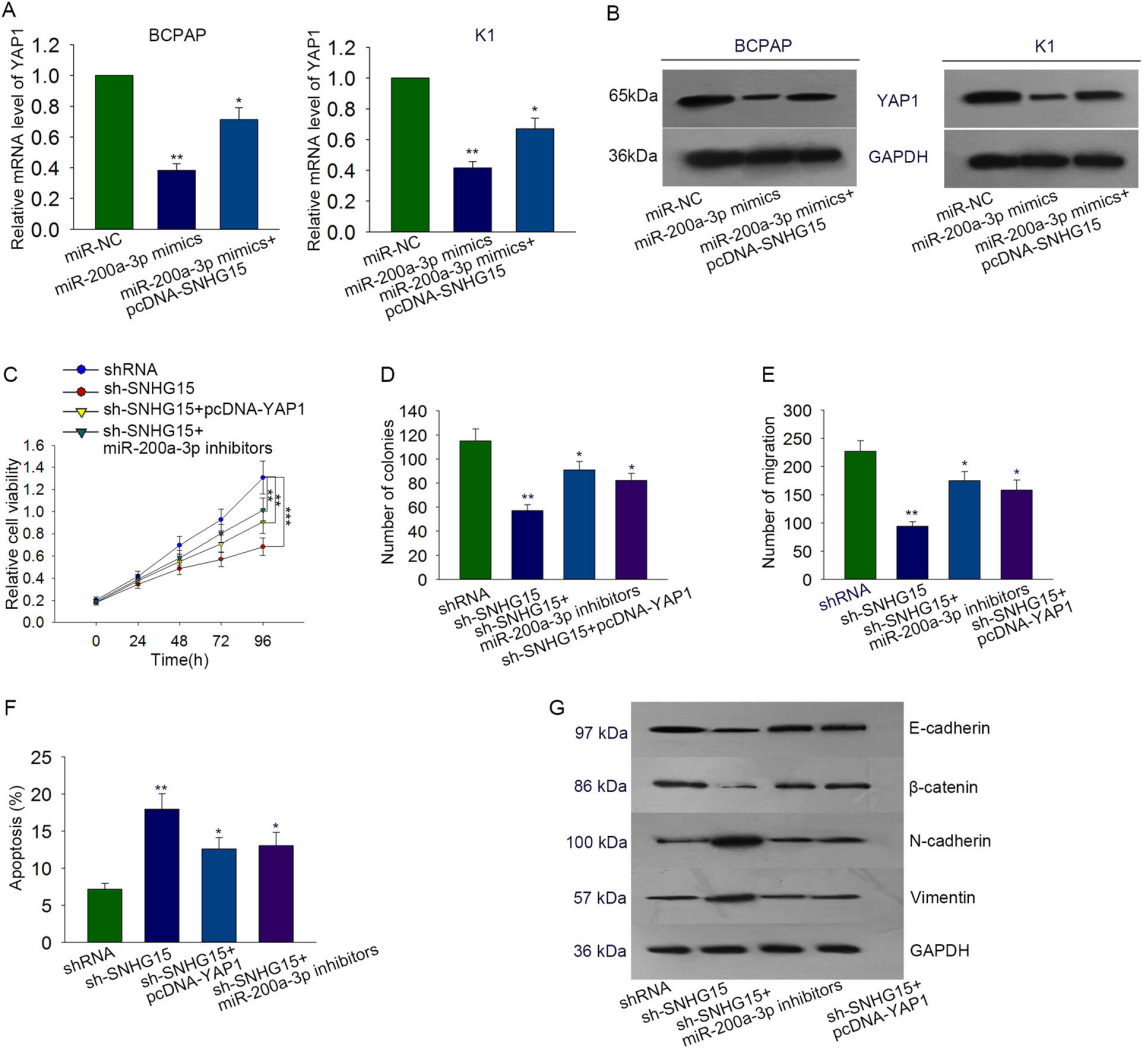
Function of SNHG15-miR-200a-3p-YAP1 axis in PTC progression. **a**, **b** mRNA level and protein level of YAP1 were measured in two PTC cells which were co-transfected with miR-200a-3p mimics and pcDNA-SNHG15. **c**, **d** MTT and colony formation assays were performed to detect proliferation ability of indicated BCPAP cell. **e** The apoptosis condition in BCPAP cell which was co-transfected with sh-SNHG15, miR-200a-3p inhibitors, and pcDNA-YAP1. **f**, **g** The metastasis and EMT process of indicated PTC cell were separately determined with transwell assay and western blot assay. *P < 0.05, **P < 0.01, ***P < 0.001

To analyze the regulatory relationship among SNHG15, miR-200a-3p, and YAP1, both mRNA level and protein level of YAP1 were measured in two PTC cells which were co-transfected with miR-200a-3p mimics and pcDNA-SNHG15. As presented in Fig. 7a, b, the decreased levels of YAP1 caused by miR-200a-3p was rescued by pcDNA-SNHG15. To determine the biological function of SNHG15-miR-200a-3p-YAP1 axis in PTC progression, rescue assays were designed and performed in BCPAP cells. According to the result of MTT and colony formation assays, decreased cell proliferation induced by sh-SNHG15 was recovered by transfecting with miR-200a-3p inhibitors or pcDNA-YAP1 (Fig. 7c, d). The increased apoptosis caused by sh-SNHG15 (*P* = 0.003, *t* = 8.911) was reduced by miR-200a-3p inhibitors (*P* = 0.03, *t* = 3.380) or pcDNA-YAP1 (*P* = 0.01, *t* = 4.699) (Fig. 7e). Meanwhile, sh-SHNG15-induced migration inhibition (*P* = 0.006, *t* = −8.414) was reversed by miR-200a-3p inhibitors (*P* = 0.011, *t* = −5.554) or pcDNA-YAP1 (*P* = 0.017, *t* = −5.193) (Fig. 7f). The inhibitory effect of sh-SNHG15 on EMT progress was reversed by miR-200a-3p inhibitors or pcDNA-YAP1 (Fig. 7g). Based on all data, we confirmed that SNHG15 acted as a ceRNA to modulate PTC progression via miR-200a-3p/YAP1 axis.

### Upregulation of SNHG15 inactivated Hippo signaling pathway

**Fig. 8:**
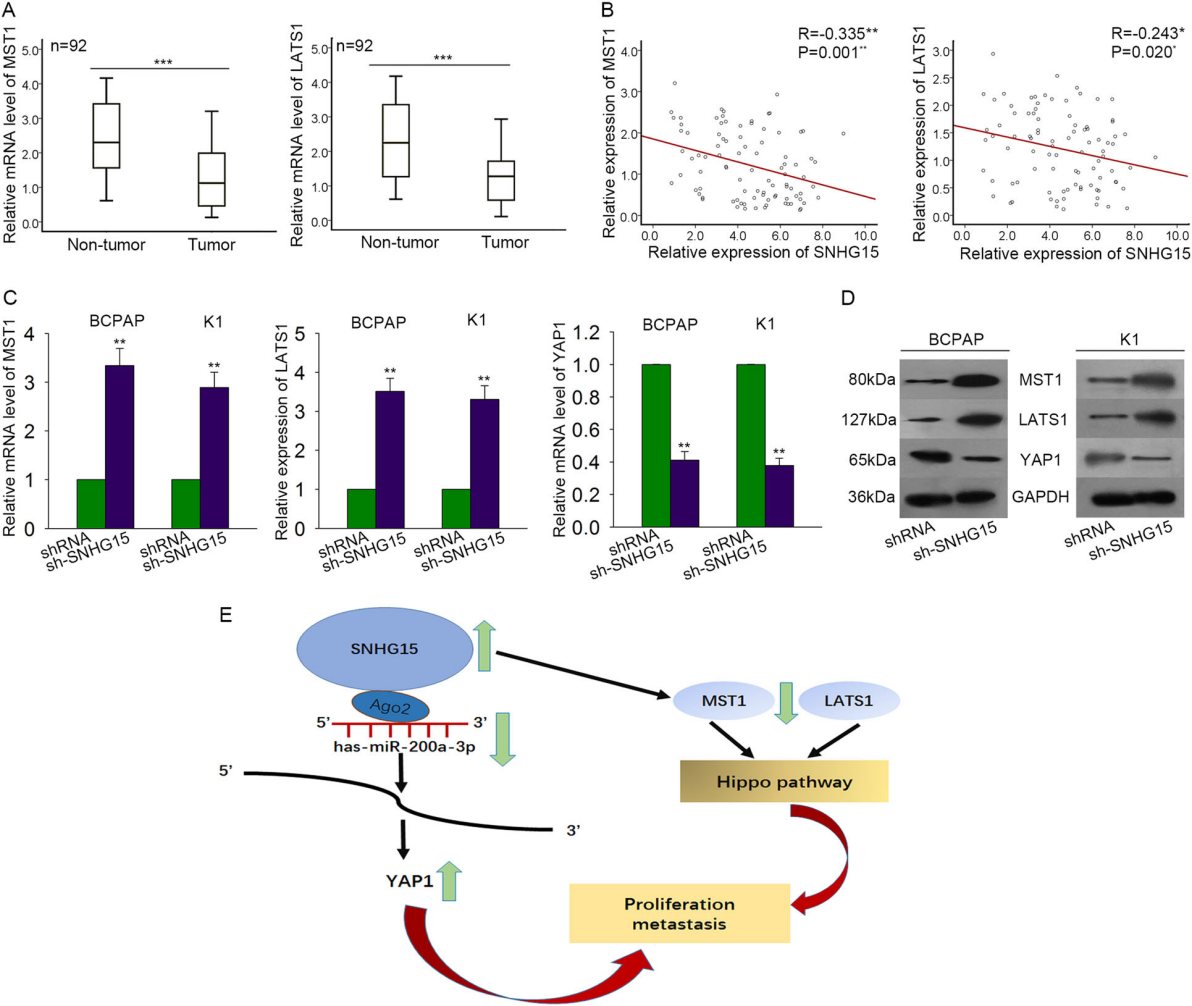
SNHG15 inactivated Hippo signaling pathway through upregulating YAP1. **a** The expression levels of MST1 and LATS1 were examined in both non-tumor tissues and PTC tissues. **b** The correlation between MST1/LATS1 and SNHG15 was analyzed. **c**, **d** Both mRNA levels and protein levels of MST1/LATS1/YAP1 were tested in SNHG15-downregulated PTC cells. **e** The mechanism diagram was generated to illustrate the mechanism of SNHG15-miR-200a-3p-YAP1-Hippo pathway in PTC. *P < 0.05, **P < 0.01, ***P < 0.001

The previous study has reported that inactivation of Hippo pathway led to downregulation of MST1/LATS1 and upregulation of YAP1. Here, we hypothesized that SNHG15 might inactivate Hippo signaling pathway. Firstly, the expression levels of MST1 and LATS1 were examined in both non-tumor tissues and PTC tissues. Both of them were downregulated in PTC tissues (Fig. 8a). The negative correlation between MST1/LATS1 and SNHG15 was analyzed (Fig. 8b, *P* = 0.001, *P* = 0.020). Next, both mRNA levels and protein levels of MST1/LATS1/YAP1 were tested in SNHG15-downregulated PTC cells. As a result, MST1 (*P* = 0.008, *t* = −11.426; *P* = 0.009, *t* = −10.407) and LATS1 (*P* = 0.006, *t* = −13.024; *P* = 0.008, *t* = −11.416) were upregulated, while YAP1 (*P* = 0.003, *t* = −11.416; *P* = 0.002, *t* = 24.306) was downregulated (Fig. 8c, d). Therefore, we confirmed that SNHG15 inactivated Hippo pathway in PTC. Taken all together, we confirmed that SNHG15 improved PTC progression through miR-200a-3p/YAP1/Hippo pathway (Fig. 8e).

## Discussion

Based on the previous studies, the role of lncRNAs has been widely revealed in human cancers. LncRNAs can regulate tumorigenesis through multiple molecular mechanisms. It is widely acknowledged that lncRNAs can exert oncogenic function in human cancers through acting as a ceRNA. For instance, lncRNA HOTTIP facilitates tumorigenesis of esophageal squamous carcinoma through exerting as a ceRNA of HOXA13^24^; lncRNA HCAL acts as a ceRNA to motivate cell proliferation and metastasis in hepatocellular carcinoma^25^; lncRNA MIR31HG drives tumorigenesis of pancreatic ductal adenocarcinoma through functioning as a ceRNA^26^. In this study, we aim to study a novel ceRNA model in PTC. LncRNA SNHG15 has been reported in human malignant tumors for its oncogenic function^17–19,21,27^. This study aims to investigate the specific mechanism and function of SNHG15 in PTC. The strong expression of SNHG15 was detected in PTC tissues and cells by qRT-PCR analysis. Moreover, the prognostic value of SNHG15 was verified through using Kaplan–Meier method. Hence, we confirmed the research value of SNHG15 in PTC. To further investigate the influences of SNHG15 dysregulation on PTC cell activities, loss-of-function assays were conducted in two PTC cells lines which hold the highest level of SNHG15. According to the results of functional assays, we knew that knockdown of SNHG15 inhibited cell proliferation and metastasis and induced cell apoptosis. Therefore, SNHG15 exhibited oncogenic property in PTC.

Previous studies have validated that lncRNAs can reverse the function of their target miRNAs in human cancers^28–33^. Here, we hypothesized that SNHG15 might act as a ceRNA by binding with a certain miRNA. The localization of SNHG15 was firstly identified in PTC cells. It was uncovered that SNHG15 is mainly located in the cytoplasm of PTC cells. Thus, we identified that SNHG15 regulated gene expression at the post-transcriptional level, indicating the potential ceRNA role of SNHG15 in PTC cells. Next, mechanism experiments, such as bioinformatics analysis, RIP assay, luciferase reporter assay, and pull down assay were utilized to find the target miRNA of SNHG15 in PTC cells. As a result, miR-200a-3p was identified to be the target miRNA of SNHG15. The negative correlation between miR-200a-3p and SNHG15 was analyzed and identified with Spearman’s correlation analysis. Accordingly, we confirmed that SNHG15 could negatively regulate miR-200a-3p by binding with miR-200a-3p. As a subgroup of non-coding RNA, miRNAs have been reported in malignant tumors^34–38^. In this study, we examined the expression pattern of miR-200a-3p in different tissues and cells. As a result, miR-200a-3p was down-expressed in PTC tissues and cell lines. Similarly, YAP1 was found to be the target of miR-200a-3p. YAP1 was found to be positively modulated by SNHG15 in PTC cells.

YAP1 was an oncogene which had been reported in various tumors^39–43^. Moreover, YAP1 was famous as the downstream gene of Hippo signaling pathway. Therefore, we hypothesized that SNHG15 could inactivate Hippo signaling pathway. To verify our hypothesis, we examined the levels of MST1 and LATS1 (the core factors of Hippo pathway) in PTC samples and normal samples. They were all downregulated in PTC tissue samples. Furthermore, SNHG15 was negatively correlated with MST1/LATS1 in PTC tissues. The mRNA level and protein level of MST1/LTA1 were negatively regulated by SNHG15 in PTC cell lines. The experimental results suggested that SNHG15 could inactivate Hippo pathway. Finally, rescue assays were carried out in BCPAP cells to demonstrate the function of SNHG15-miR-200a-3p-YAP1-Hippo pathway in PTC. In summary, SNHG15 improved PTC progression through modulating miR-200a-3p/YAP1-Hippo pathway. Our experimental findings might provide the potential therapeutic pathway for PTC.
